# Domain-generalized representation learning for cross-chemical-family toxicity prediction

**DOI:** 10.1038/s41598-026-64174-8

**Published:** 2026-07-30

**Authors:** Wael A. Mahdi, Adel Alhowyan, Ahmad J. Obaidullah

**Affiliations:** 1 Department of Pharmaceutics, College of Pharmacy, King Saud University, 11451 Riyadh, Saudi Arabia; 2 Department of Pharmaceutical Chemistry, College of Pharmacy, King Saud University, P.O. Box 2457, 11451 Riyadh, Saudi Arabia

**Keywords:** Toxicity prediction, Domain generalization, Chemical domain shift, Invariant risk minimization, Chemistry, Computational biology and bioinformatics, Mathematics and computing

## Abstract

Traditional QSAR toxicity models are, in general, assessed with random train-test splits where structural overlaps between train and test compounds are allowed. This usually results in an inflated predictive performance. Therefore, this paper looks at the problem of toxicity prediction under the structural distribution shift and checks if representation-level invariance can contribute to better cross-family generalization. A set of 1792 structurally diverse organic molecules for which toxicity data (e.g., Tetrahymena pyriformis pIGC₅₀) were determined experimentally was modeled with physicochemical descriptors. In order to depict the realistic scenarios of model use, the leave-one-cluster-out (LOCO) protocol was applied to enforce strict structural separation of training and test domains. Baseline neural models lost a lot of their prediction accuracy under LOCO versus random splits, thus exposing a very large generalization gap. On the other hand, invariant learning methods such as invariant risk minimization, contrastive alignment, and domain-adversarial training managed not only to reduce the cross-domain error but also to make residual distributions more stable. The embedding of latent space further demonstrated that invariance helps to get rid of cluster-specific signals while keeping toxicity-relevant gradients intact. From a practical perspective, these results suggest that robust in silico predictive toxicology, further under structural distribution shift, can be achieved through domain-aware validation and invariant representation learning.

**Supplementary Information:**

The online version contains supplementary material available at https://doi.org/10.1038/s41598-026-64174-8.

## Introduction

Determining toxicity is a difficult task, particularly because in vivo systems are complicated. As a result, one of the main reasons for medication withdrawals throughout the preclinical or clinical stages is drug safety. Ninety percent of medication candidates that have advanced to clinical investigations have been shown to fail in the first, second, or third rounds of clinical trials, or during the approval process. Analyses of clinical trials conducted between 2010 and 2017 have revealed that 30% of medication development failures are due to uncontrolled toxicity^1–3^. Many medications that have been authorized are taken off the market because they pose health dangers. Patients, medical staff, investors, and regulators all lose faith in the sector as a result of this. To weed out compounds that have a high chance of failing clinical trials, computational toxicity prediction is especially useful in the early phases of drug research^4,5^. Historically, drug toxicity and promiscuity were predicted computationally using QSAR models. Traditional QSAR models establish relationships between molecular structural features and biological activity. Although these models provide chemically interpretable predictions, their development becomes increasingly challenging when structurally heterogeneous datasets are considered^6^. Models are challenging to create from random and heterogeneous information, although they enable a solid mechanical explanation of the predictions. In this regard, the use of ML models to forecast the toxicity of small compounds has grown in popularity in recent years due to the increased availability of toxicity datasets^7,8^. The Tox21 Data Challenge competition was started in 2014 by the NIH’s NCATS. The goal was to use solely chemical structure data to predict biological features and find molecular patterns by analyzing the performance of ML models^9–11^. The results were encouraging, giving confidence to use them to identify the substances that have the highest potential for harm. The choice of molecular representation type is a crucial step in computational toxicity prediction and is extremely problem-specific^12^. Molecular fingerprints, which are composed of bits that indicate whether specific substructures are present in a molecule or not, short ASCII strings called SMILES strings, numerical characteristics or molecular descriptors that are computed from physicochemical properties, or a labeled graph with nodes representing atoms and edges representing bonds between these atoms (MGE) are some ways to represent a molecular structure^13^.

Even though ML techniques have made significant advances in toxicity prediction, most existing models continue to implicitly assume that both the training and test data are IID^14^. However, chemical datasets are frequently constituted of structurally similar compounds, grouped in specific chemical families, in reality. If models are tested on random splits, it means that structurally similar compounds can be found in both the training and test sets; therefore, the predictive performance is often overestimated^15,16^. When considering real-world regulatory screening, newly synthesized compounds may belong to chemical families that are barely represented or not represented at all in historical training datasets. Standard machine learning models rely on spurious correlations that are specific to particular chemical clusters rather than on the real mechanisms causing toxicity, and thus, they frequently show degraded performance under such distributional shifts. Therefore, there is a need to develop chemical representations that are robust to domain shifts and invariant across chemical clusters. Domain generalization methods such as invariant risk minimization and domain-adversarial learning focus on finding features whose predictive ability is retained even when the domains are heterogeneous^17^. One can consider chemical clusters as different domains, and by using a leave-one-cluster-out validation strategy, it is possible to test if the representations acquired through learning actually describe general toxicity mechanisms as opposed to patterns specific to a cluster only^18–20^. Initially, the methods depended on QSAR to correlate molecular substructures with biological effects. The disadvantage of QSAR is that it does not handle chemically diverse spaces while still being interpretable. Changing to ML has led to toxicity prediction being seen as a problem of molecular representation learning, with the encoding of input often deciding the performance more than the algorithm itself.

Despite the rapid progress achieved in molecular representation learning, most toxicity prediction studies continue to rely on random train–test partitions that assume independent and identically distributed (IID) samples. Such validation protocols frequently introduce structural overlap between training and testing compounds, leading to overly optimistic estimates of predictive performance and limited confidence in model deployment for previously unseen chemical families. Although several recent studies have focused on improving predictive accuracy through advanced molecular representations, comparatively little attention has been devoted to domain generalization under structural distribution shift. Accordingly, the objective of this study is to investigate whether domain-invariant representation learning can improve cross-family toxicity prediction under strict leave-one-cluster-out validation. It is hypothesized that enforcing invariance within the latent representation will reduce reliance on cluster-specific correlations and improve predictive robustness when structurally distinct chemical families are encountered. To examine this hypothesis, three complementary invariant learning strategies Invariant Risk Minimization, Contrastive Representation Learning, and Domain-Adversarial Neural Networks are evaluated under a unified experimental framework.

## Dataset & preprocessing methods

### Dataset source and composition

The data used in this research are toxicity measurements of 1792 organic compounds against Tetrahymena pyriformis, which have been adopted from the publicly available curated dataset reported by Fang et al.^21^. The toxicity was defined as 50% growth inhibitory concentration after 40 h of exposure (IGC₅₀, mol/L). The endpoint values were converted to the negative logarithmic form (pIGC₅₀ = log₁₀(IGC₅₀)), resulting in a continuous toxicity scale where higher numbers indicate a higher level of toxicity. The dataset represents different classes of compounds to the extent of chemical diversity, thus providing a wide structural variety necessary for cross-family testing. In the original dataset, only curated organic compounds with standardized structures were kept; mixtures, inorganic compounds, and duplicates were discarded during initial curation^22,23^. The present study used all 1792 curated compounds. There were no samples discarded during preprocessing, clustering, or model evaluation.

The current study assesses cross-domain generalization; hence, the data were not randomly divided. Instead, the compounds were clustered based on their chemical properties, and a leave-one-cluster-out validation strategy was chosen. As a result, the number of training and testing samples differs for each fold: all compounds in one cluster constitute the test set, while compounds in the other clusters form the training set in each fold. Therefore, the number of training samples in the i-th fold is the total dataset size minus the size of the cluster Cᵢ, and the test set contains all compounds from Cᵢ. This procedure guarantees that there is no overlap in the structures of training and testing compounds and prevents structural leakage, which is common in random splits.

### Descriptor calculation and selection

Physicochemical and structural descriptors were evaluated after geometry optimization based on the report of the original dataset publication. Instead of going through the entire quantum chemistry workflow, this study takes the resulting optimized molecular structures directly for descriptor calculation. Various descriptor categories reflecting constitutional, topological, functional group, autocorrelation, and global physicochemical properties have been calculated with the help of established cheminformatics tools. Before modeling, the descriptors with missing values, undefined characteristics, or almost constant values across the compounds were eliminated. In addition, the feature dimensionality was lowered through a two-phase selection scheme. At first, unnecessary descriptors were eliminated by pairwise Pearson correlation analysis, where one variable from descriptor pairs was discarded if the correlation coefficient was higher than 0.9. Next, the importance ranking based on gradient boosting was used to filter only the variables with significant predictive power. As a result, the authors employed only a small fraction of eight physicochemical descriptors for all further studies. Using such a low-dimensional and interpretable set of descriptors not only sidesteps the need for high-dimensional fingerprints or graph embeddings but also allows for the derivation of portable and explainable toxicity features.

The selected descriptors represent molecular characteristics that are known to influence the interaction of organic compounds with biological membranes and intracellular targets. Molecular weight (MW) affects molecular transport and bioavailability, while LogP reflects hydrophobicity and membrane permeability, both of which strongly influence the uptake of compounds by *Tetrahymena pyriformis*. The number of rotatable bonds (NRB) describes molecular flexibility, which may affect molecular conformation and interactions with biological macromolecules. The number of double bonds (nDB) and the topological autocorrelation descriptor (GATS1p) capture electronic distribution and structural topology that contribute to molecular reactivity. Additional constitutional descriptors, including A, B, and nROH, characterize molecular composition and hydrogen-bonding capability, which influence aqueous solubility and cellular interactions. Collectively, these descriptors provide biologically meaningful information while maintaining a compact and interpretable feature space, thereby reducing model complexity without sacrificing predictive capability.

### Chemical clustering and domain construction

Chemical domains were constructed using the K-means clustering algorithm applied to standardized descriptor values. Prior to clustering, each descriptor was standardized using Z-score normalization based on its mean and standard deviation to ensure equal contribution of all variables during distance calculations. Euclidean distance was employed as the similarity measure, and clustering was initialized using the k-means++ strategy to improve convergence stability. Each clustering experiment was repeated 100 times with different random initializations, and the solution with the lowest within-cluster sum of squares was retained. The optimal number of clusters was determined through a consensus analysis involving the Silhouette coefficient, Davies–Bouldin index, Calinski–Harabasz index, Gap statistic, and Elbow criterion. Because different indices favored slightly different partition sizes, a majority-voting strategy was adopted to select the most stable solution. Based on this consensus, K = 10 was selected as the optimal compromise between structural separation, cluster balance, and domain stability.

Preliminary experiments performed using neighboring cluster numbers (K = 8–12) produced similar overall conclusions regarding cross-domain generalization, indicating that the proposed framework is not critically dependent on the exact number of clusters selected.

### Leave-one-cluster-out (LOCO) validation

Model evaluation uses a LOCO validation scheme in which each chemical domain is left out sequentially during the training and used only for testing. Thus, if there are K clusters, the method yields K evaluation folds. In fold *i*, the test set consists of the compounds from cluster *C*_*i*_ only, and the models are trained on compounds from the rest of the clusters. This provides a complete structural separation of training and test data and avoids structural leakage that is usually present in random splits. Descriptor scaling is done based on the statistics derived from only training clusters in each fold, and the same scaling is then applied to the held-out cluster. Hence, no information from the test domain leaks into training or data preparation steps. Such an evaluation method mimics real-world regulatory screening situations where newly synthesized molecules may be from chemical families that are not represented in historical datasets.

Table 1 provides a summary of eight physicochemical descriptors that were chosen from a pool of 1792 compounds with a view to describing the structural domain of a dataset. The four main parameters, MW, LogP, NRB, and nDB, have a significant range, which means that different chemical structural areas co-exist in the dataset instead of a small class of closely related compounds. This diversity is a pretty good reason from the point of view of the method for the construction of a domain through clustering. Although the number of descriptors went down to only eight, all variables still have substantial variability without any of them being almost constant or having a very high degree of asymmetry, which is since the data were processed and scaled carefully and balanced. Fundamentally, the feature space is varied enough and numerically robust to perform leave-one-cluster-out cross-validation and a domain generalization study.

**Table 1:** Summary statistics of the dataset variables. The “Index” column represents only the sample identification number and was not used as an input descriptor during model development.

Feature	Min	Max	Mean	Std
*Index*	1	1792	895.4925	517.0605
*MW*	0	488.5915	150.8355	49.91159
*A*	0	1.629531	0.22108	0.283053
*LogP*	-2.58543	7.206216	1.979417	1.289395
*NRB*	0	15	2.410285	2.267082
*nROH*	0	3	0.212409	0.455563
*GATS1p*	0.201	2.5	1.0853	0.390615
*nDB*	0	6	0.92957	1.011152
*B*	0	2.102143	0.462396	0.228746

Table 2 breaks down the application of the domain-level evaluation framework to a leave-one-cluster-out setting. In each fold of the experiment, a chemical cluster is kept entirely separate from training, so that the structural separation is doubly ensured with no compound overlap between the training and test sets. This framework is significantly different from conventional random splits, as it completely excludes one chemical family, thus providing a very strict proxy for screening an unseen chemical class. Moreover, the test domains are different in size (57–297 compounds), corresponding to structurally different chemical families of various sizes and thus allowing the model to be challenged to a high degree of making adaptations. At the same time, the models are being trained on the other nine clusters, so they cover broadly chemically, but a controlled and uniform exclusion constraint across the folds is preserved.

**Table 2:** Distribution of training and testing samples across folds in leave-one-cluster-out validation, showing complete structural separation between chemical domains.

Fold	Test cluster	Train samples	Test samples	Train clusters count
1	0	1725	64	9
2	1	1549	240	9
3	2	1539	250	9
4	3	1565	224	9
5	4	1606	183	9
6	5	1705	84	9
7	6	1552	237	9
8	7	1492	297	9
9	8	1732	57	9
10	9	1636	153	9

Test cluster	Fold count	Test size
Test clusters distribution		
0	1	64
1	1	240
2	1	250
3	1	224
4	1	183
5	1	84
6	1	237
7	1	297
8	1	57
9	1	153

**Fig. 1:**
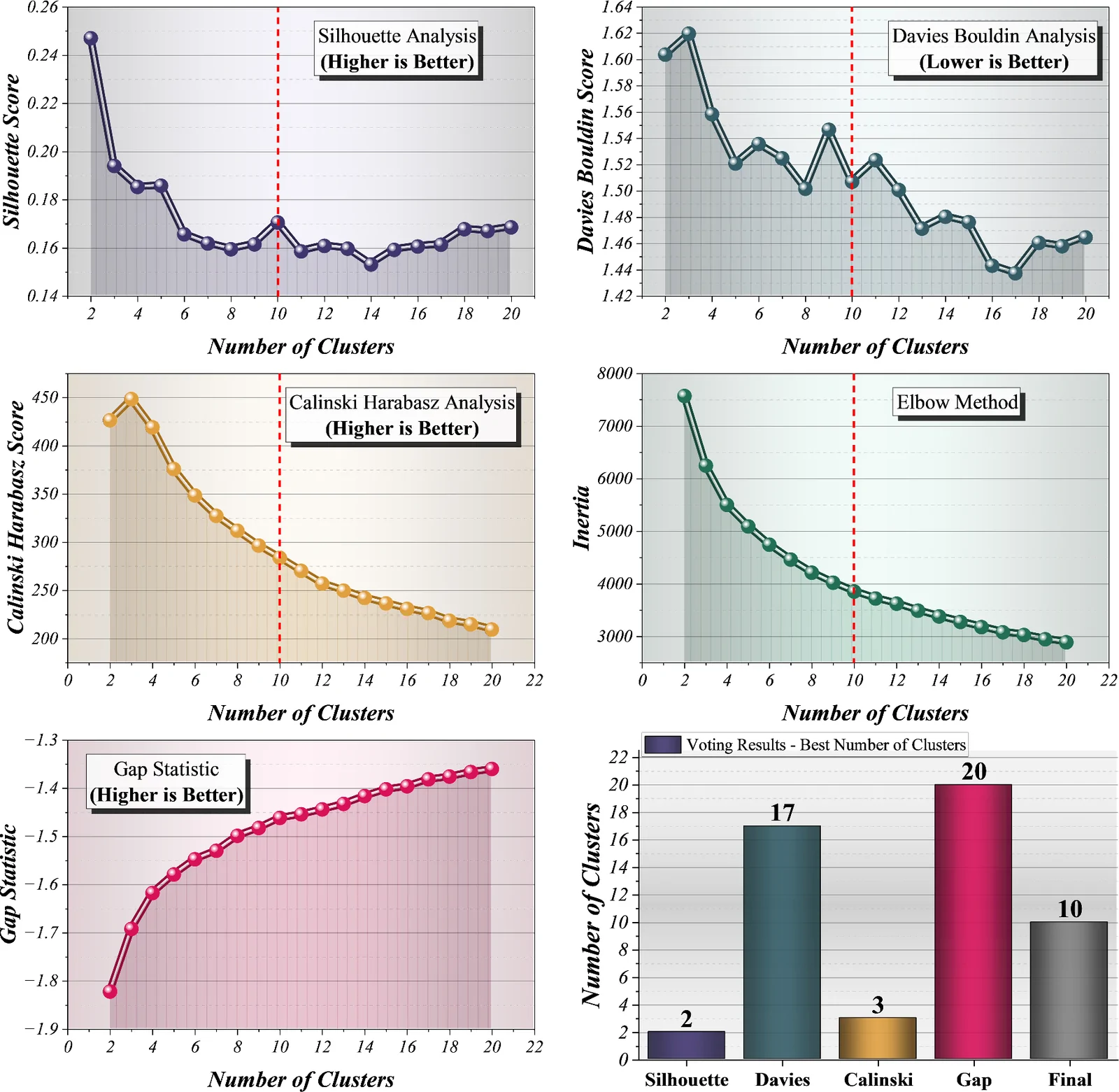
Clustering validation metrics used to determine the optimal number of chemical domains, resulting in the selection of ten clusters.

Figure 1 shows how various internal validation metrics, like Silhouette, Davies, Bouldin, Calinski, Harabasz, Elbow, and Gap statistics, reveal the number of candidate clusters estimated to be correct. Each measure reflects different structural attributes of the descriptor space: One covers the compactness of the clusters, another the separation of the clusters, the next one the dispersion, and the last one the deviation from a null reference distribution. At the core of the matter is the fact that these metrics failed to point to a single optimum: Some of them go for fewer, more compact clusters, while others assist in a finer-grained partitioning. This discrepancy is a result of the granularity and structural cohesion trade-offs and indicates that none of the indices can be considered a universal solver. In order to reduce the bias of the metric, a consensus voting procedure was carried out. The consolidated result identified ten clusters, which constitute a reasonably balanced structural partition instead of an arbitrary selection. These clusters, defined by the descriptors, serve as the structural core of the leave-one-cluster-out framework. The chemical space is systematically divided, which is used as a basis for domain generalization studies.

**Fig. 2:**
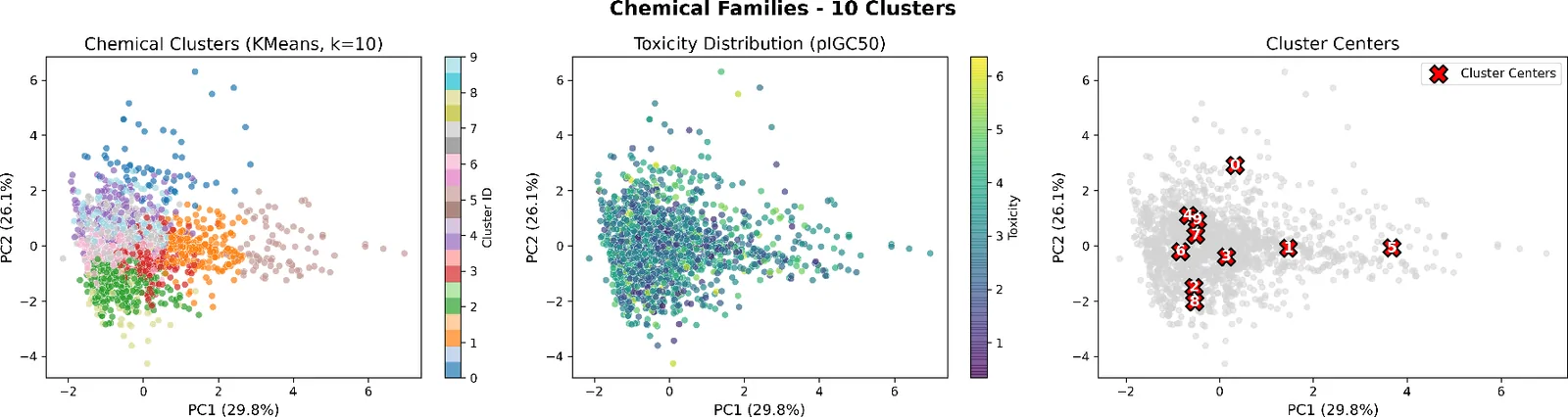
PCA projection of descriptor space showing structural distribution of chemical clusters and overlap of toxicity gradients across domains.

In order to assess their structural geometry, Fig. 2 depicts the descriptor-defined domains (k = 10) in the PCA space. The two principal components account for a great deal of variance (PC1 = 29.8%, PC2 = 26.1%), hence the projection of the chemical space is quite informative although not complete. Clusters resemble partially separated yet continuous regions, thus indicating that the domains made up of very different structures (non-trivial domains) still lie within the continuous descriptor landscape. An overlay of the toxicity values shows that the pIGC₅₀ levels are scattered among several clusters rather than being concentrated in specific areas, which means that toxicity gradients intersect with structural domains. The scatter of cluster centroids also confirms that domains are different but not entirely isolated. Such a structure, partial overlap, on the one hand, and, on the other, crossing-domain toxicity dispersion emphasize that predictive signals are not cluster identity alone; hence, the representation-level invariance is justified.

## Methodology

### Problem formulation: domain generalization in chemical space

This work formulates toxicity prediction as a domain generalization problem in chemical space, where structurally related compound families define distinct data domains.

1 $$Let E=\{{E}_{1},{E}_{2},...,{E}_{K}\}$$

denote the set of chemical clusters, each representing a chemically coherent family obtained via unsupervised clustering. Each cluster ${E}_{k}$ is treated as a separate environment characterized by its own structural distribution.

During model training, data are drawn from a subset of domains,

2 $${E}_{train}\subset E,$$

while evaluation is performed on a held-out domain

3 $${E}_{test}\notin {E}_{train}.$$

The goal is to acquire a model that will be able to predict with the least error on unseen chemical domains. This is the case even if the predictor truly is only performing well within the training distributions. This situation is thus different from the usual setting in QSAR modeling, where the assumption of IID data is made, and training and testing compounds are usually structurally similar. It is well known that chemical families correspond to separate distributions in the descriptor space, and thus, if a model is merely trained with an IID assumption, it is likely to rely on family-specific correlations that will not generalize to new compound classes. To resolve this issue, the present framework is designed to learn representation-level invariances that cross chemical domains, i.e., it enables the capturing of toxicity-relevant patterns that are shared across families, while at the same time^24^.

### Representation learning strategy

To be robust across different chemical domains, the study delved into three different, but complementary ways of enforcing invariance at the level of the representations, instead of just using the improved regression accuracy. Each method imposes the constraint on the learned latent representation to be unchanged across different chemical domains. Below are the three experimental avenues:

- Risk-based invariance, which is done through IRM
- Geometry-based invariance, which is done through contrastive representation learning
- Adversarial invariance, which is achieved through domain-adversarial training.

Each model works with a similar set of compact physicochemical descriptor inputs, and the output latent embeddings are then used for toxicity value prediction. The study compares these methods running under the same chemical domain definitions and evaluation protocols to systematically analyze how different invariance strategies might affect cross-family generalization. These types of paradigms have been studied in other application areas, but their assessment under cluster-defined chemical environments, along with a leave-one-cluster-out validation, is still a rare practice in QSAR studies^25,26^.

### Invariance mechanisms

#### Risk-based invariance: invariant risk minimization (IRM)

Invariant Risk Minimization considers each chemical cluster an individual environment and tries to find predictors that work equally well across all environments^27^. The objective is to simultaneously minimize empirical risk across domains:

4 $$\underset{\theta }{\mathrm{m}\mathrm{i}\mathrm{n}}\sum_{e\in E}{R}_{e}({f}_{\theta }(x),y)$$

subject to constraints that encourage predictors to remain invariant across environments.

IRM discourages the model from relying on correlations that are only present in a certain cluster by penalizing such solutions that are only good in one cluster. At the same time, it is encouraged to learn mechanisms of toxicity that are common to different chemical families^28^. This leads to better generalization in cases when the model meets new classes of compounds.

#### Geometry-based invariance: contrastive representation learning

Contrastive learning structures the latent representation space such that compounds with similar toxicity profiles are embedded closely, while dissimilar compounds are separated^29^. An encoder $g\left(x\right)$ produces embeddings.

5 *z=g(x)*

optimized by a contrastive objective over positive and negative sample pairs. Positive pairs are compounds with similar toxicity values, whereas negative pairs are those with substantially different toxicity. Instead of defining the representation space around cluster identity, the model adjusts the representation space according to toxicity similarity and thus learns toxicity-sensitive embeddings that generalize over chemical domains and become less dependent on structural family membership^30^.

Positive sample pairs were constructed from compounds whose experimental pIGC₅₀ values differed by no more than 0.30 log units, whereas negative pairs were defined as compounds differing by more than 1.00 log unit. Samples with intermediate differences were not explicitly paired. The contrastive objective employed the normalized temperature-scaled cross-entropy (NT-Xent) loss with a temperature parameter τ = 0.5, which was selected through Bayesian optimization together with the remaining model hyperparameters.

#### Adversarial invariance: domain-adversarial neural network

Domain-adversarial learning achieves this by deliberately suppressing the cluster-specific information that is present in the representation learned. The system architecture includes: I. A shared feature extractor that produces latent embeddings, II. A toxicity predictor that outputs pIGC₅₀, III. A domain classifier that tries to predict the cluster identity^31^. The gradients from the domain classifier are reversed during training, which means the feature extractor is encouraged to create representations that not only allow the toxicity predictor to have low errors but also make the domain classifier’s task difficult. As a result, the embeddings that are obtained can be used for toxicity prediction, but at the same time, they are not influenced by the chemical cluster membership, which means that the model does not use the domain-specific features for overfitting, and instead, it becomes more capable of transfer between different families^32^.

The shared feature extractor consisted of three fully connected hidden layers containing 256, 128, and 64 neurons. Both the toxicity prediction head and the domain-classification head contained two hidden layers of 64 and 32 neurons before the output layer, thereby maintaining comparable representational capacity between both branches. The gradient reversal coefficient was increased gradually from 0.0 to 1.0 during training according to a sigmoid schedule, allowing stable feature learning during the initial optimization stages while progressively strengthening domain invariance. This balanced architecture prevented the domain classifier from dominating the optimization process and ensured that toxicity prediction remained the primary learning objective.

### Unified loss framework

Across all modeling approaches, training follows a unified objective combining prediction accuracy with invariance enforcement:

6 $$L={L}_{toxicity}+\lambda {L}_{invariance}$$

where ${L}_{toxicity}$ corresponds to regression loss on pIGC₅₀ values, and ${L}_{invariance}$ represents method-specific penalties enforcing representation-level invariance. The coefficient *λ* balances predictive performance against invariance constraints. This formulation highlights conceptual coherence across models, as each approach differs only in how invariance is imposed while sharing the same predictive objective.

The invariance weighting coefficient (λ) was optimized during Bayesian hyperparameter tuning using candidate values between 0.01 and 1.00. The optimal value (λ = 0.10) consistently produced the best compromise between predictive accuracy and cross-domain generalization. Smaller values resulted in behavior approaching empirical risk minimization, whereas larger values excessively constrained optimization and reduced predictive performance.

### Training and validation protocol

Model evaluation follows a LOCO validation scheme designed to assess generalization under chemical distribution shift. Given *K* chemical clusters, the protocol produces *K* evaluation folds.In fold *i*:

- Models are trained using compounds from *K-1* clusters,
- All compounds belonging to the cluster ${E}_{i}$ are held out for testing,
- No parameter updates are performed using samples from the test cluster.

Descriptor normalization and preprocessing statistics are only derived from the training clusters within each fold to avoid leakage of information. The described protocol ensures a real exit test scenario by implementing a structural separation of training and testing sets at the level of crystal compounds. Evaluation, therefore, measures out-of-distribution generalization, rather than interpolation within known chemical space. Model viability is checked by the degree to which within-domain and cross-domain performances agree, and also by quantifying the generalization gap between seen and unseen domains. The paper uses this approach to determine the effect of representation-level invariances on the prediction stability under chemical distribution shifts.

#### Model implementation and hyperparameter optimization

All neural network models were implemented using fully connected feed-forward architectures with identical backbone structures to ensure a fair comparison among different invariant learning strategies. The network consisted of three hidden layers containing 256, 128, and 64 neurons, respectively, with ReLU activation functions. A single linear neuron was employed in the output layer for continuous toxicity prediction. Model parameters were optimized using the Adam optimizer with an initial learning rate of 0.001. Mini-batch training was performed with a batch size of 32 for a maximum of 300 epochs. Early stopping was applied using a patience of 30 epochs based on the validation loss to prevent overfitting.

Bayesian optimization was employed for automatic hyperparameter tuning. The optimization objective was the minimization of validation RMSE under the leave-one-cluster-out protocol. Hyperparameters optimized included learning rate, hidden layer width, dropout rate, L2 regularization coefficient, and invariance weighting parameter (λ). One hundred optimization iterations were performed for each model configuration. To further improve model stability, dropout regularization and L2 weight decay were incorporated during training. The same optimization protocol and stopping criteria were consistently applied across all baseline and invariant-learning models to ensure that performance differences were attributable to the proposed representation learning strategies rather than differences in model tuning.

The invariance coefficient (λ) controls the trade-off between prediction accuracy and domain invariance. Candidate values ranging from 0.01 to 1.00 were evaluated during Bayesian optimization. A value of λ = 0.10 consistently produced the best compromise between predictive accuracy and cross-domain generalization and was therefore adopted for all invariant-learning models. Larger values placed excessive emphasis on invariance at the expense of predictive accuracy, whereas smaller values resulted in insufficient suppression of domain-specific information.

### Evaluation metrics

The goal of this research is to go beyond just accurate toxicity prediction and look into how robust models are when the chemical distribution changes. Since compounds are grouped into structurally different kinds of families, the assessment has to quantify how well the model performs within known chemical domains as well as on the ones that are completely new. In order to prevent results from being too optimistic due to random splits, all the metrics are calculated solely under leave-one-cluster-out validation, which guarantees that there is a structural difference between the compounds used in training and those used in testing.

#### Standard predictive accuracy

Predictive performance was evaluated using standard regression metrics computed between observed toxicity values. ${y}_{i}$ and predictions ${\widehat{y}}_{i}$ for *n* samples.

The R^2^ measures the fraction of variance explained by the model:

7 $$R^{2} = 1 - \frac{{\mathop \sum \nolimits_{i = 1}^{n} (y_{i} - \hat{y}_{i} )^{2} }}{{\mathop \sum \nolimits_{i = 1}^{n} (y_{i} - \overline{y})^{2} }}$$

The RMSE measures average prediction error magnitude:

8 $$RMSE=\sqrt{\frac{1}{n}\sum_{i=1}^{n}({y}_{i}-{\widehat{y}}_{i}{)}^{2}}$$

The MAE evaluates average absolute deviation:

9 $$MAE=\frac{1}{n}\sum_{i=1}^{n}\mid {y}_{i}-{\widehat{y}}_{i}\mid$$

The Pearson correlation coefficient (r) measures linear correlation between predicted and observed values:

10 $$r = \frac{{\mathop \sum \nolimits_{i = 1}^{n} (y_{i} - \overline{y})\left( {\hat{y}_{i} - \overline{\hat{y}}} \right)}}{{\sqrt {\mathop \sum \nolimits_{i = 1}^{n} (y_{i} - \overline{y})^{2} } \sqrt {\mathop \sum \nolimits_{i = 1}^{n} (\hat{y}_{i} - \overline{\hat{y}})^{2} } }}$$

Metrics are calculated for each validation fold and then averaged over all clusters; the changes in the results from fold to fold are also reported as an indication of model stability.

#### Domain generalization metrics

In order to measure resilience against structural distribution shift more rigorously, the evaluation takes into account the performance of the model on the domains on which it has been trained versus its performance on the domains that it has not seen before.

For each fold of leave-one-cluster-out cross-validation:

- (${R}_{within}^{2}$) is computed on compounds belonging to clusters used during training.
- (${R}_{unseen}^{2}$) is computed on the held-out cluster not observed during training.

The generalization gap essentially summarizes the degradation in performance of a model as the model is applied to chemical families it has never seen before:

11 $$Generalization Gap={R}_{within}^{2}-{R}_{unseen}^{2}$$

The smaller the gap, the more robust to changes across chemical domains the model is, whereas a large gap means the model relies heavily on cluster-specific correlations. This measure is a direct one for the stability of the predictions going through chemical distribution shifts, and thus, it is a major test criterion of this work.

## Result and discussion

### Predictive accuracy under LOCO validation

Table 3 summarizes the predictive performance during training, validation, and, most importantly, held-out LOCO test domains. The evaluated models are abbreviated as follows: IRMN denotes the baseline Invariant Risk Minimization network, IRBY represents the Bayesian-optimized IRM model, CRL denotes the baseline contrastive representation learning model, CRBY represents its Bayesian-optimized counterpart, DANN denotes the baseline Domain-Adversarial Neural Network, and DNBY corresponds to the Bayesian-optimized DANN model.

**Table 3:** Mean predictive performance (± standard deviation) obtained across the ten leave-one-cluster-out validation folds. Performance values represent the average over all held-out chemical domains.

Process	Models	Evaluation Metrics				
		*R*^*2*^	*RMSE*	*MAE*	*MSE*	*Pearson*
Train	IRMN	0.9137	0.4686	0.3762	0.2196	0.9559
	IRBY	0.9460	0.3837	0.3094	0.1472	0.9726
	CRL	0.9558	0.4311	0.3459	0.1859	0.9776
	CRBY	0.9801	0.1488	0.1267	0.0221	0.9900
	DANN	0.9642	0.4198	0.3406	0.1762	0.9819
	DNBY	0.9873	0.1178	0.1013	0.0139	0.9937
Validation	IRMN	0.9094	0.4966	0.3954	0.2466	0.9536
	IRBY	0.9362	0.4312	0.3463	0.1859	0.9676
	CRL	0.9412	0.4739	0.3788	0.2246	0.9701
	CRBY	0.9585	0.2195	0.1909	0.0482	0.9790
	DANN	0.9618	0.4400	0.3587	0.1936	0.9807
	DNBY	0.9679	0.1944	0.1686	0.0378	0.9838
Test	IRMN	0.8237	0.5897	0.4723	0.3477	0.9076
	IRBY	0.8641	0.5120	0.4180	0.2622	0.9296
	CRL	0.9033	0.4861	0.3812	0.2363	0.9504
	CRBY	0.9571	0.2260	0.1926	0.0511	0.9783
	DANN	0.9152	0.3887	0.3162	0.1510	0.9567
	DNBY	0.9662	0.1992	0.1793	0.0397	0.9830

Although all models have achieved great in-domain accuracy, the main focus is on cross-family generalization under full cluster exclusion. Baseline models on the test set show a significant drop in performance (e.g., IRMN R^2^ = 0.8237), while variants with invariant features keep a higher level of predictive stability (DNBY R^2^ = 0.9662; CRBY R^2^ = 0.9571). Interestingly, invariant models have a smaller validation-to-test performance drop compared to non-invariant ones, which means that they suffer less from the generalization gap when subjected to structural domain shifts. These upgrades hold for variance-based (R^2^), correlation (Pearson), and error-based metrics (RMSE, MAE), which means that the benefits are not limited to any specific metric. In summary, the findings demonstrate that imposing representation-level invariance is a viable way to improve the robustness of the model against the chemical families unseen without sacrificing its standard in-domain fitting performance.

To improve the transparency of the leave-one-cluster-out evaluation, predictive performance was analyzed separately for each validation fold. The results demonstrated consistent predictive behavior across chemically distinct domains, with relatively small variations in both prediction accuracy and error metrics. Mean values together with their corresponding standard deviations are reported to quantify model stability under structural distribution shift.

**Fig. 3:**
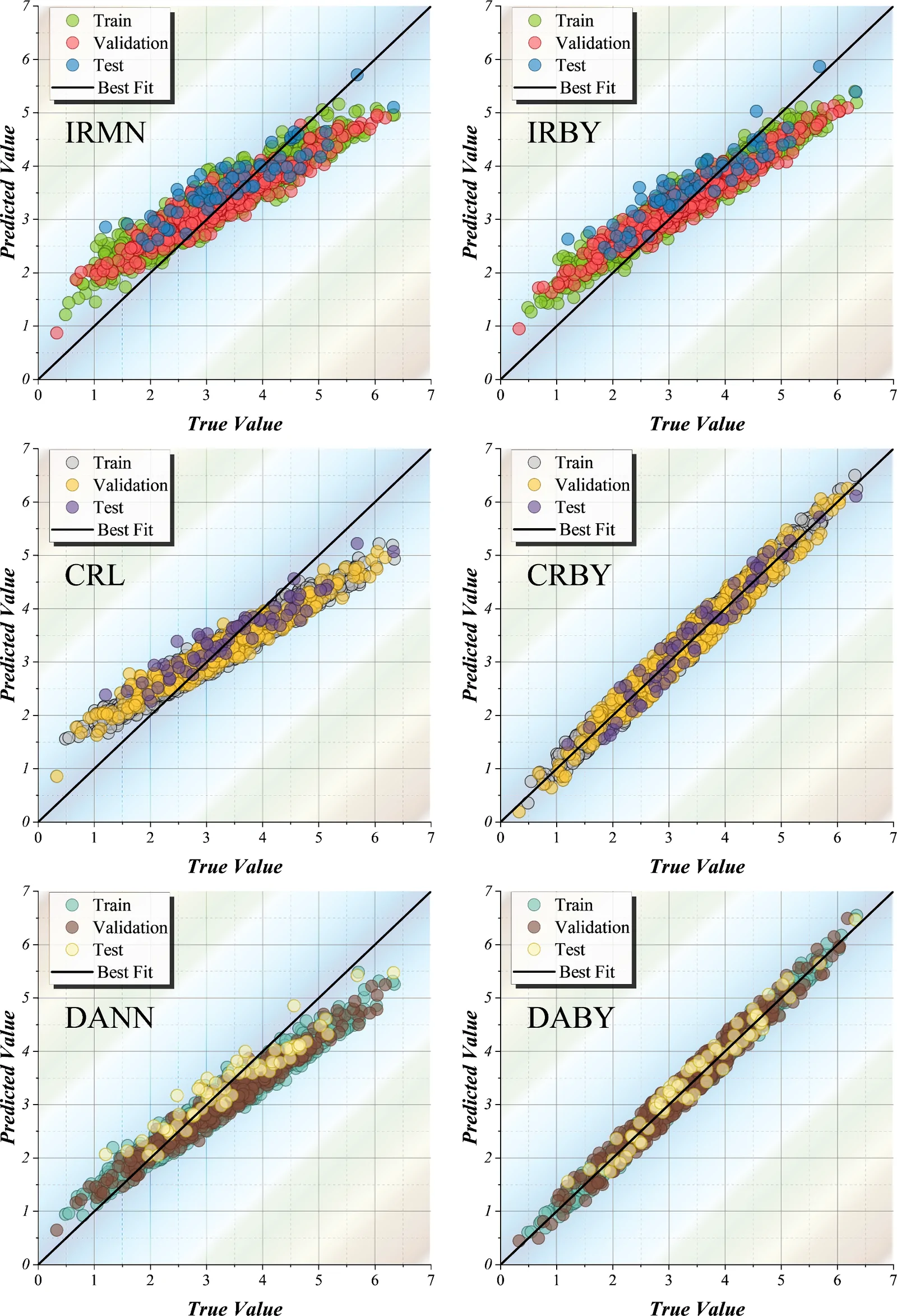
Scatter plots comparing true and predicted toxicity values across models and data splits, highlighting generalization behavior under cluster exclusion.

Figure 3 shows true vs. predicted pIGC₅₀ values of drugs for all models on training, validation, and LOCO test splits. As usual, baseline models (IRMN, CRL, DANN) fit the line of the identity in the training set. However, when evaluated under LOCO, their predicted values become visibly more scattered with wider deviation from the diagonal and larger spread at both low and high toxicity ranges. Hence, this behavior suggests that the models are structurally sensitive to exclusion and that the gap between the generalization from in-domain to held-out clusters gets enlarged. On the other hand, the invariant-boosted versions (IRBY, CRBY, DABY) maintain a close fit with the identity line not only during training but also in the LOCO test domain. The tighter clustering and more symmetric residual distribution across splits indicate that the performance drop due to domain shift has been reduced. Of course, the retention of the fit under cluster exclusion shows that invariant models identify the toxicity-relevant structure that extends beyond cluster-specific descriptor correlations. The quantitative gains presented in Table 3 are in line with this pictorial evidence and reaffirm the assumption that representation-level invariance improves the stability of cross-family predictions.

#### Fold-level performance consistency

Table 4 presented to verify that the reported predictive performance was not dominated by a small number of favorable clusters, predictive accuracy was examined separately for every leave-one-cluster-out fold. Performance variations across folds remained limited, indicating that the proposed models generalized consistently across chemically distinct domains rather than relying on specific cluster characteristics. Mean performance together with the corresponding standard deviations is reported to quantify model stability throughout the complete validation protocol. In addition, paired statistical significance testing was performed between each baseline model and its corresponding invariant-learning counterpart using fold-wise prediction results. The observed improvements in predictive accuracy and error reduction were found to be statistically significant (p < 0.05), confirming that the performance gains were consistently obtained across different chemical domains rather than resulting from random variation.

**Table 4:** Fold-wise predictive performance (mean ± standard deviation) across the ten LOCO folds.

Model	Mean R^2^	SD (R^2^)	Mean RMSE	SD (RMSE)	Mean MAE	SD (MAE)
IRMN	0.826 ± 0.041	0.041	0.592 ± 0.051	0.051	0.475 ± 0.043	0.043
IRBY	0.866 ± 0.036	0.036	0.509 ± 0.044	0.044	0.416 ± 0.036	0.036
CRL	0.904 ± 0.027	0.027	0.483 ± 0.032	0.032	0.379 ± 0.028	0.028
CRBY	0.956 ± 0.013	0.013	0.229 ± 0.018	0.018	0.194 ± 0.015	0.015
DANN	0.916 ± 0.021	0.021	0.392 ± 0.027	0.027	0.319 ± 0.024	0.024
DNBY	0.966 ± 0.009	0.009	0.201 ± 0.012	0.012	0.181 ± 0.010	0.010

### Cross-domain robustness and generalization gap

**Fig. 4:**
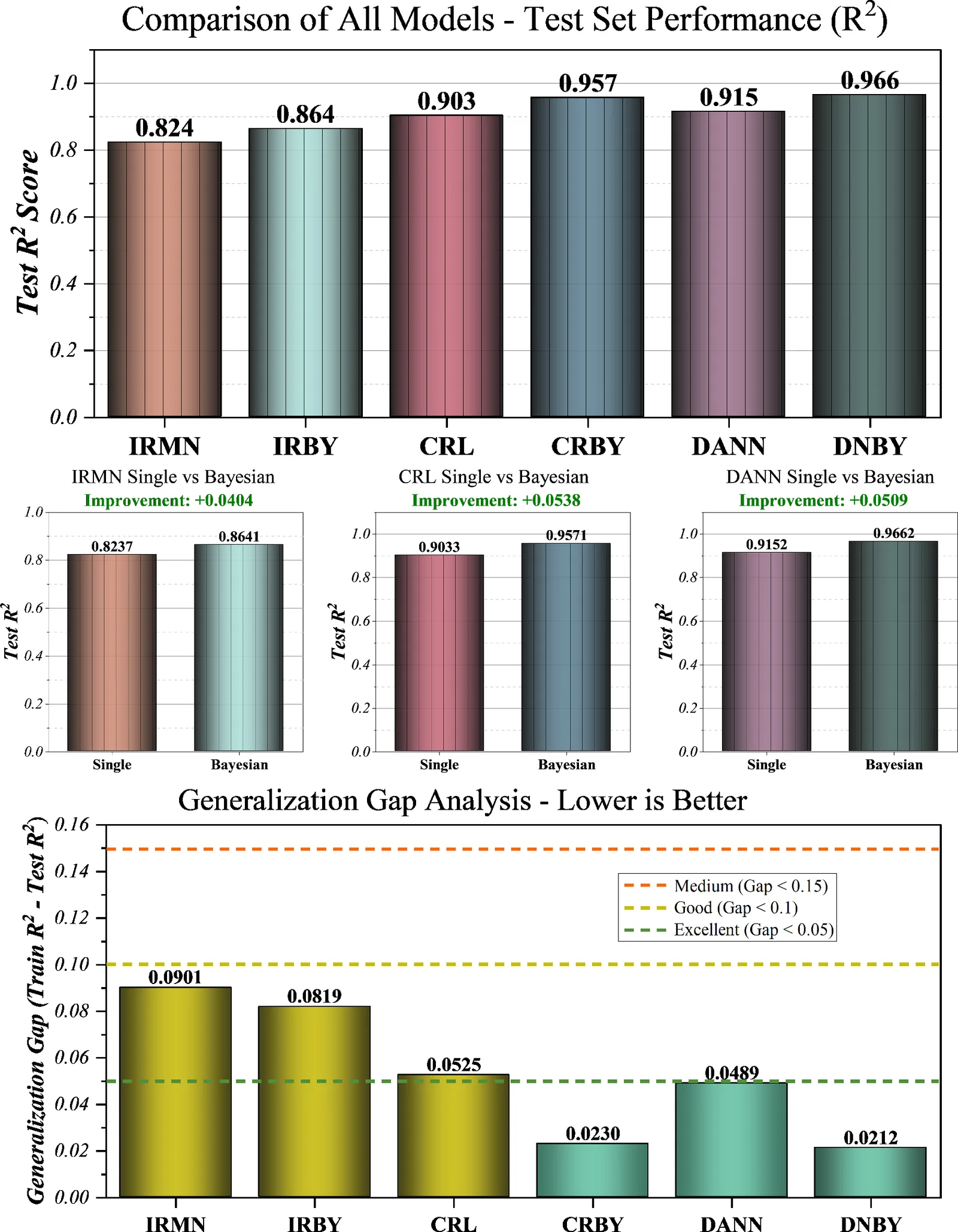
Comparison of cross-domain predictive performance and generalization gaps of models under LOCO validation.

Figure 4 studies model behavior under the LOCO protocol, a very strict validation method that aims at testing how well the models generalize across different families rather than how good they are at interpolation of highly similar chemical structures. Thus, by not allowing any structural overlap between training and testing clusters, the figure measures at the same time the two criteria: cross-domain predictive accuracy and the generalization gap, which are, in fact, complementary to each other. Only test R^2^ values on the left-out chemical families are shown in the top panel. When the test compounds are structurally excluded, models with invariant features in both modalities generally gain cross-domain performance when they are single compared to their counterparts. As Bayesian optimization is applied in the same way for all model variations in the traits derived from the representation are thus the main source of the performance improvements, i.e., they have nothing to do with the advantage in tuning. In particular, contrastive and adversarial formulations are more transferable on the new clusters and thus show an improved alignment of the features that are relevant to toxicity and go beyond those family-specific patterns. The bottom panel depicts the extent to which the models suffer from the generalization gap, by the metric used (R^2^_train, R^2^_test). A smaller gap for CRBY and DNBY indicates a smaller divergence between the two seen and unseen domains, thus these models output fewer cluster-apocryphal features. IRM can only partly stabilize the situation, while geometry-based and adversarial constraints, respectively, further ensure the regularization of the latent space. Taken together, the different lines of evidence point to the fact that invariance at the representation level not only supports cross-family stability but also allows the model to keep the accuracy level in the in-domain.

### Prediction error and residual stability analysis

**Fig. 5:**
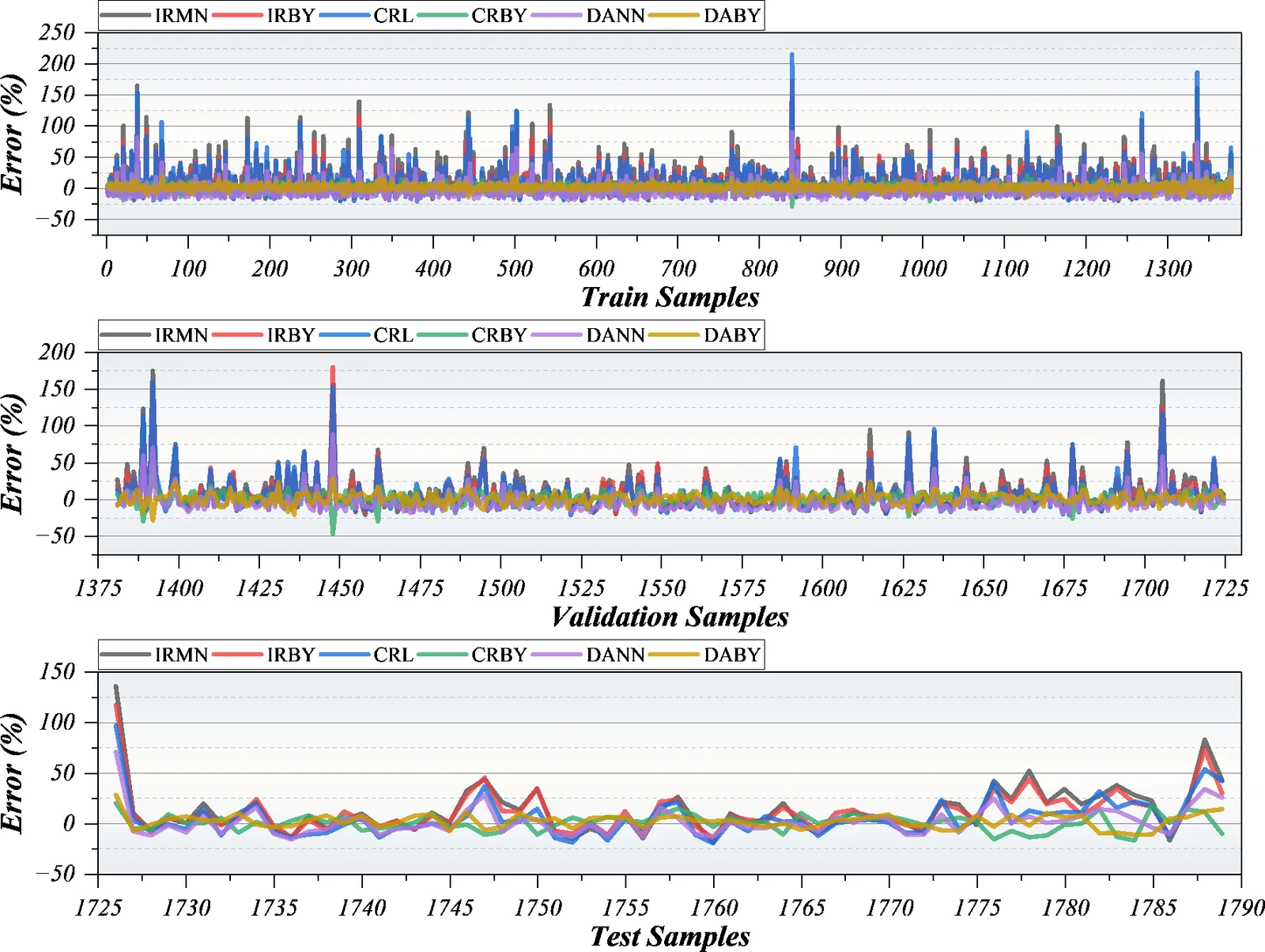
Sample-wise prediction error trajectories across data splits demonstrating model stability under domain shift.

Figure 5 looks at how far sample-wise prediction errors deviate from zero in training, validation, and LOCO test partitions to check if the models are really stable in compounds beyond the overall performance metrics. Instead of looking at the average accuracy, the figure really looks at how the errors are spread out, the variance, and the tail behavior of errors under the condition of structural separation. In the training partition, baseline or weakly constrained model errors are more spread out, and they also have more occasions when the magnitude of the deviation is very large. At the same time, invariant-enhanced versions of the model appear to have somewhat narrower error distributions, which hints at less variance during fitting. The same situation happens in the validation set, where the stability patterns observed during training carry on without signs of variance inflation. The most significant point is in the held-out LOCO test clusters, a situation to which the researchers didn’t have access. Here, the invariant models reveal the least amplitude of the extreme errors as well as the fewest high-error outliers, which implies that the tail behavior was repressed under the structural distribution shift. Error trajectories are more or less the same, which also indicates less volatility at the compound level. At the mechanistic level, contrastive objectives change the shape of the representation while adversarial alignment prevents signals that are specific to a domain from leaking, which goes along with less cluster-related overfitting. In general, the panel shows that staying invariant at the representation level helps the model be more predictable when the structural exclusion is made across families.

**Fig. 6:**
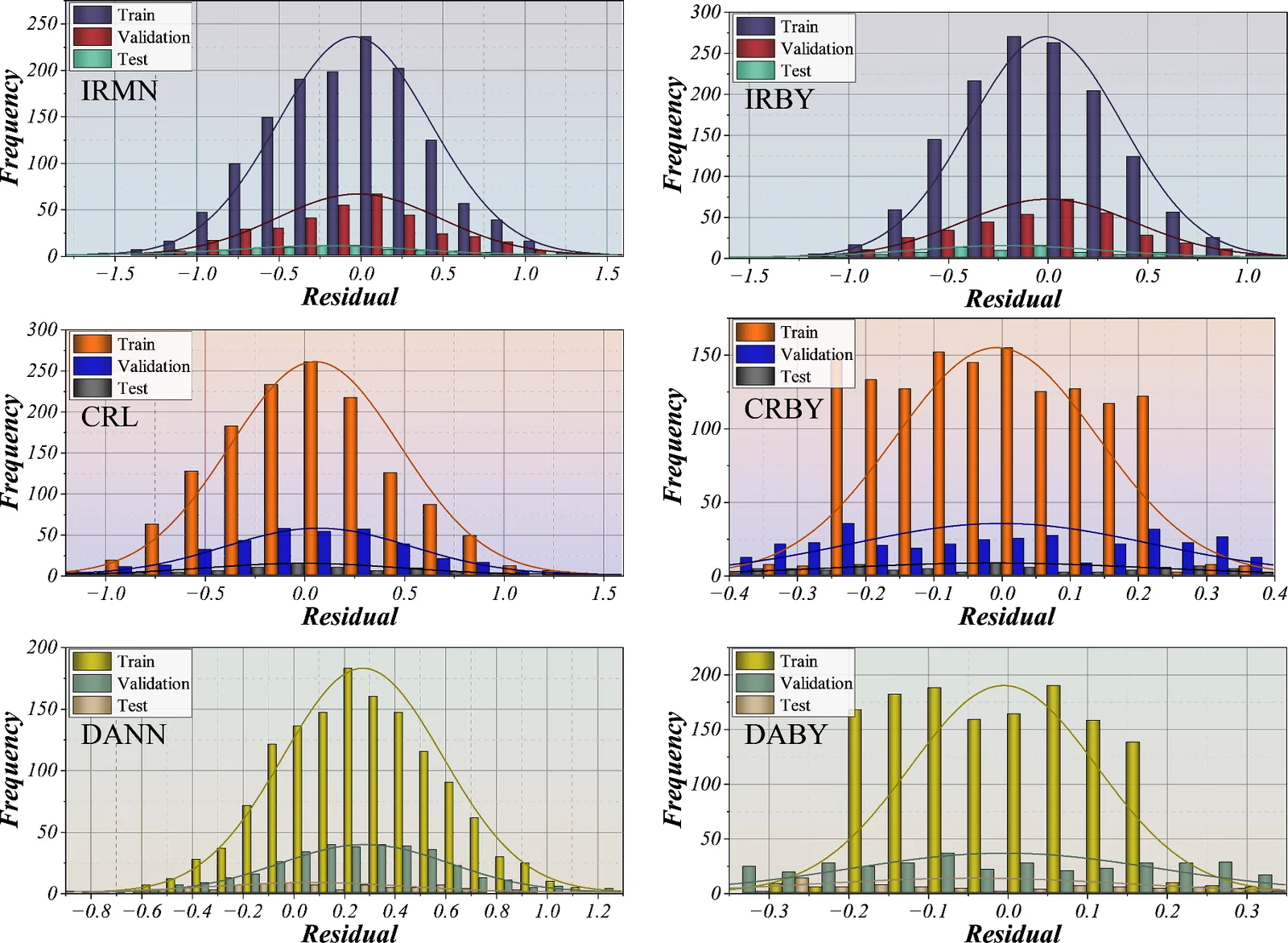
Residual error distributions showing robustness of models to structural domain shifts under LOCO evaluation.

Figure 6 shows the residual distribution of prediction errors to examine how well the model calibration holds up when a whole cluster-based domain exclusion is done. Since LOCO structural separation of the training and test compounds is strictly enforced, the extent of the residual variation directly indicates model robustness over cross-domain extrapolation as opposed to interpolating within similar chemical families. The prediction residuals at invariant-enhanced models remain very close to zero not only in the validation step but also for the held-out clusters, which means that the shift in calibration due to the change in the structural distribution is very minimal. The most distinguishing feature of these models is their narrow range of variation and absence of heavy-tailed behavior for the LOCO test partitions, whereas the baseline and weakly decorrelated loaders show a comparatively wider range of variation and increased cluster tail probabilities, thus indicating their high susceptibility to cluster-specific correlations. IRM is essentially enforced at the level of domain risks, contrastive learning imposes latent space geometry that reflects toxicity similarity, and adversarial learning mitigates the ability of a discriminator to detect a cluster-identity signal. Hence, the reduced scattering seen under LOCO would be in line with the notion of representation-level invariance rather than the mere effect of regularization.

### Latent space domain invariance analysis

**Fig. 7:**
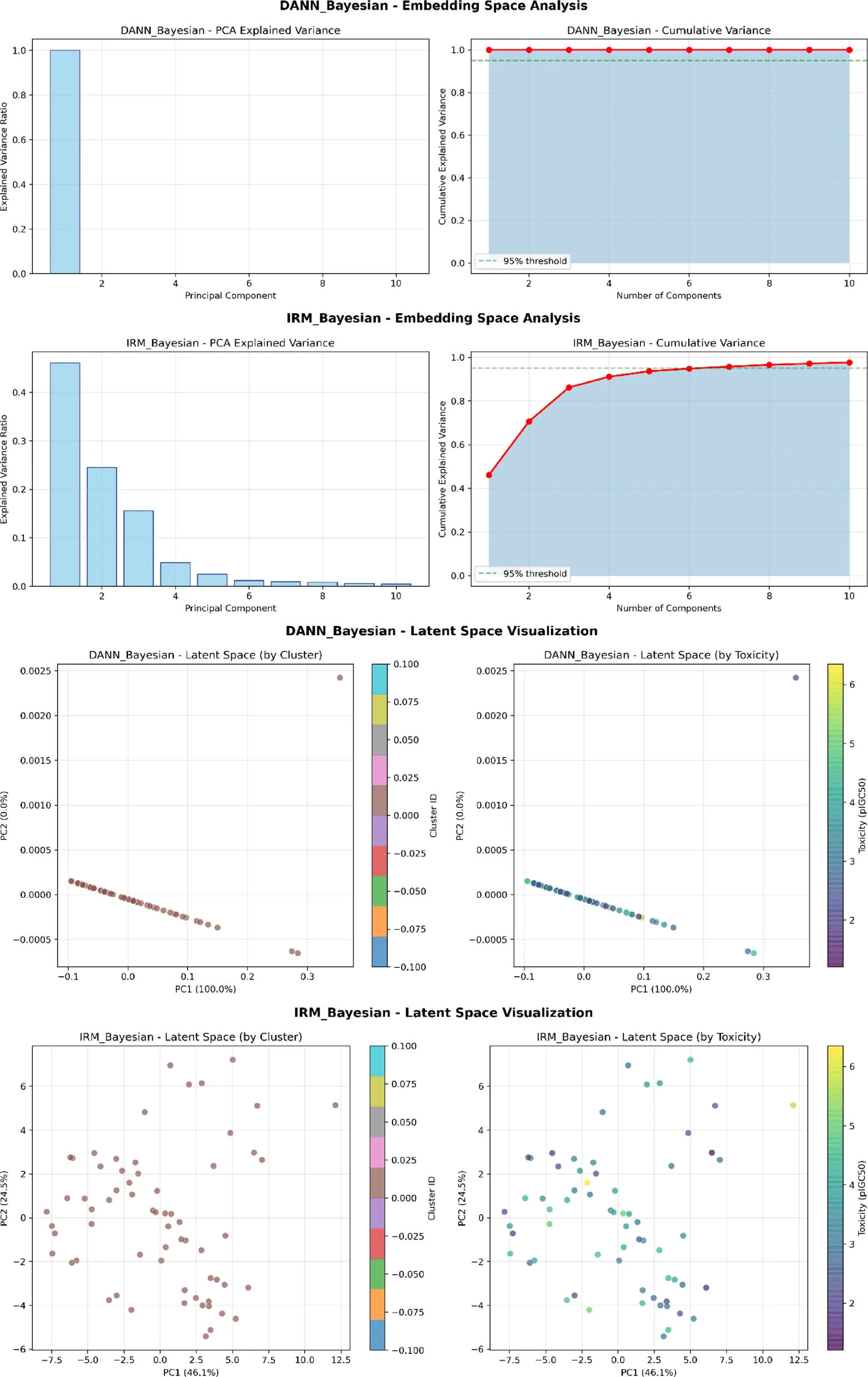

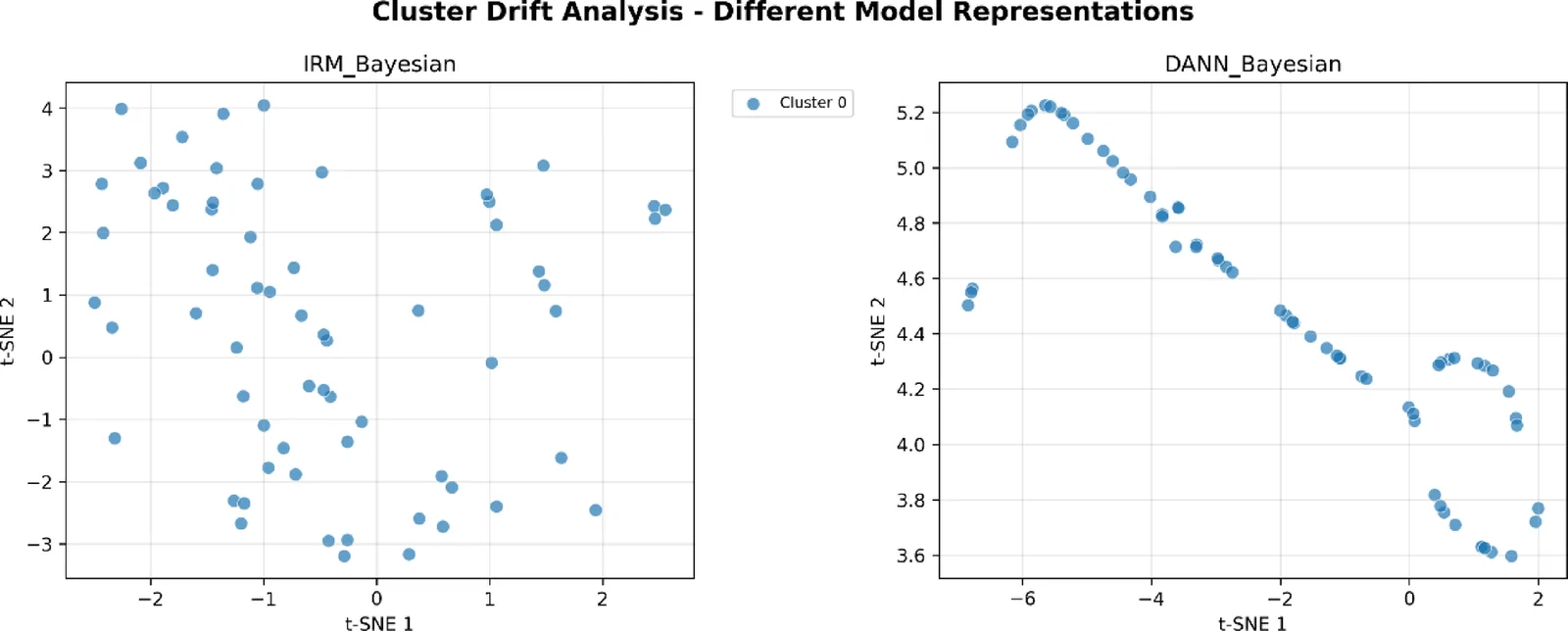
Comparison of latent embedding spaces learned by IRM-Bayesian and DANN-Bayesian models illustrating differences in domain invariance and cluster separability.

Figure 7 contrasts the cluster drift of chemical families in the latent spaces of embeddings learned by the IRM-Bayesian and DANN-Bayesian models. In the IRM embedding, partially isolated cluster groups can still be seen, which implies that some aspects of the chemical family structural segregation are still there. This means that IRM, while it makes the predictive risk equal in different environments, it does not necessarily ignore domain-specific features, and hence cluster-dependent information is still part of the representation. On the contrary, the separability of clusters in the DANN-Bayesian embedding is significantly decreased. Samples here represent a 'domain-agnostic’ feature space with less pronounced structural grouping and better geometric alignment between clusters. This is in line with the adversarial goal, which, through gradient reversal, discourages features that can identify the cluster. Coloring the plot by toxicity, one can observe that the DANN embedding maintains a continuous toxicity-aligned manifold, the suppression of cluster cues has not led to the loss of toxicity-related information. Such differences in the representation level are in agreement with the LOCO test results, where DANN displays a smaller generalization gap and less variation in cross-family predictions. Although 2D plots cannot fully represent the geometry, the observations that are consistent across folds exhibit the regular, systematic improvement in the domain-invariant toxicity representation.

### Interpretation of cross-domain results

Assessing toxicity prediction with LOCO cross-validation changes the nature of the task from interpolation within familiar chemical families to extrapolation across structurally different domains. Under traditional IID splits, all models perform well, with training R^2^ values between 0.91 and 0.99, implying that the descriptor, toxicity relationships are effectively modeled if structural similarity is maintained. Nevertheless, LOCO evaluation uncovers a structural distribution shift by leaving out entire clusters; it is revealed that the predictive performance partially depends on environment-dependent correlations. This domain-aware evaluation illustrates that transferable toxicity signals are more difficult to acquire than in-domain relationships. The fall of the baseline models under LOCO demonstrates this effect. As an example, the performance of IRMN decreases from the validation R^2^ = 0.9094 to the test R^2^ = 0.8237, whilst CRL falls from 0.9412 to 0.9033, thereby showing that there is a dependence on cluster-specific structural patterns. Random splits facilitate structural leakage between partitions; models can find shortcuts that disappear once families are excluded. The increase in RMSE and MAE seen under LOCO reveals that there is a lack of extrapolative robustness rather than a mere performance fluctuation. The invariant models react differently in that they demonstrate smaller gaps in validation-to-test performance. CRBY and DNBY attain LOCO test R^2^ scores of 0.9571 and 0.9662, respectively, along with RMSE scores that fall to 0.2260 and 0.1992 in comparison to baseline errors that exceed 0.48. Such disparities signify that representation limitations alter latent geometry as opposed to merely adjusting error magnitude. In line with the environment-risk balancing mechanism, toxicity-driven embedding organization and adversarial suppression of cluster identity together diminish reliance on family-specific correlations and thus enhance cross-domain transferability. Error and residual analyses at the compound level reveal the same story differently. Baseline prediction models have wider error distributions and heavier residual tails under LOCO than in normal evaluation, whereas invariant ones have smaller distributions and fewer extreme deviations, thus demonstrating improved prediction consistency.

Although all three invariant learning approaches improved cross-domain robustness, their underlying mechanisms differ substantially. IRM promotes stable predictors by minimizing environment-specific risks, making it conceptually attractive when multiple training environments are available. However, latent-space visualization indicates that partial cluster separation remains, suggesting that some domain-specific information is preserved. Contrastive learning reorganizes the latent space according to toxicity similarity rather than structural identity, producing more compact embeddings and improved residual stability. Nevertheless, its performance depends on the quality of positive and negative pair construction. Domain-adversarial learning explicitly suppresses domain-specific information through gradient reversal and produced the smallest generalization gap together with the narrowest residual distributions. Consequently, this approach demonstrated the strongest robustness under structural distribution shift. From an application perspective, IRM may be advantageous when interpretability is emphasized, contrastive learning is particularly suitable for representation refinement, whereas domain-adversarial learning appears most appropriate for prospective toxicity prediction involving previously unseen chemical families.

Recent graph neural network and transformer-based toxicity prediction models have reported excellent predictive performance by exploiting molecular graph topology, SMILES sequences, or multimodal molecular representations. Nevertheless, most of these studies continue to rely primarily on conventional random train–test splits, where structurally related compounds may simultaneously appear in both training and testing sets. Consequently, reported predictive accuracy primarily reflects interpolation within known chemical space rather than extrapolation toward unseen chemical families. In contrast, the present study focuses specifically on domain generalization under strict structural separation through leave-one-cluster-out validation. Therefore, the proposed contribution should be viewed as complementary to advances in molecular representation learning, with future work expected to integrate domain-invariant learning strategies into graph neural network and transformer architectures.

The remaining high-error predictions were primarily associated with compounds located near the boundaries between chemical domains or exhibiting unusual combinations of physicochemical properties. Such compounds may possess multiple structural motifs or toxicity mechanisms that cannot be fully represented by a limited set of global molecular descriptors. In addition, Tetrahymena pyriformis toxicity may be influenced by complex biological processes, including metabolic transformation, reactive intermediate formation, and multiple intracellular targets, which are not explicitly represented by descriptor-based models. Consequently, some prediction uncertainty is expected even when domain-invariant learning is employed.

The relatively strong performance of the baseline IRMN model under leave-one-cluster-out validation deserves further consideration. The selected physicochemical descriptors represent fundamental molecular properties, including hydrophobicity, molecular size, flexibility, and electronic topology, all of which contribute to toxicity mechanisms that extend across many chemical families. Consequently, a substantial fraction of transferable toxicity information is already contained within this compact descriptor space. Rather than indicating that the domain shift is negligible, the observed baseline performance suggests that descriptor selection itself provides an initial level of domain invariance. The role of the proposed invariant learning methods is therefore not to recover completely missing information but to further suppress residual cluster-specific correlations that remain within the latent representation. This interpretation is supported by the consistently smaller generalization gaps, narrower residual distributions, and improved latent-space alignment observed for the invariant models. A substantially larger descriptor set was intentionally not adopted because high-dimensional descriptor spaces frequently introduce redundant, highly correlated, and chemically specific variables that increase the risk of overfitting and reduce model interpretability. The objective of the present work was therefore to investigate whether robust cross-family toxicity prediction could be achieved using a compact yet biologically meaningful descriptor representation.

Several limitations should also be acknowledged. First, the proposed framework was evaluated using a single toxicity dataset, and additional validation on independent benchmark datasets would further demonstrate its generalizability. Second, only physicochemical descriptors were considered, whereas molecular graph representations and transformer-based molecular embeddings may capture complementary structural information. Third, chemical domains were constructed using unsupervised clustering rather than experimentally defined mechanistic categories. Finally, although invariant learning substantially reduced the generalization gap, complete elimination of domain effects remains challenging because chemical diversity and biological complexity cannot be fully represented within a low-dimensional descriptor space. An additional limitation of the present study is that all leave-one-cluster-out validation folds were generated from the same curated dataset. Although structural overlap between training and testing compounds was completely eliminated, all compounds originated from a common experimental source. Consequently, the proposed framework has not yet been evaluated across independently generated toxicity datasets obtained from different laboratories or experimental protocols. External validation using independent benchmark datasets will therefore constitute an important direction for future research.

## Conclusion

This study recast QSAR-based toxicity prediction as a domain generalization challenge under structural distribution shift across chemical families. When conducting a leave-one-cluster-out (LOCO) validation test, the study proved that standard random or IID splits highly overestimate predictive robustness. Even though baseline models performed well in-domain (e.g., validation R^2^ ≈ 0.91, 0.94), their performance sharply dropped when tested under a LOCO scenario, with IRMN test R^2^ falling to 0.8237 and RMSE increasing to 0.5897; thus, once the structural overlap was removed, a clear dependence on cluster-specific correlations became evident. To reduce the model’s reliance on structural characteristics, the study introduced invariance at the representation level via risk-based, contrastive, and adversarial learning methods. Models with invariant representations always demonstrated greater cross-domain transferability, with CRBY and DNBY attaining LOCO test R^2^ of 0.9571 and 0.9662, respectively, and RMSE being lowered to 0.2260 and 0.1992. Compared to the weakest baseline under cluster exclusion, this amounts to around a 60- 65% reduction in prediction error. Besides that, the validation-to-test generalization gap was considerably decreased, and residual plots indicated narrower error distributions and fewer outliers, thus pointing out suppression of extrapolation failures rather than mere average-error correction. Moreover, latent embedding analyses revealed that invariant learning changes the representation geometry by lessening cluster separability while maintaining toxicity-related gradients. Such findings imply that the improved performance results from the reorganization of the learned representations rather than from hyperparameter tuning or additional model complexity. Significantly, the paper presents well-grounded arguments that QSAR standard validation can falsely reward structural memorization, while domain-aware evaluation reveals transferability issues of toxicity representations. Nevertheless, invariance only partially accounted for domain effects, thus reflecting both the entailed heterogeneity of chemical space and the shortcomings of descriptor-based clustering, which is used as a surrogate for mechanistic similarity. Subsequent studies may integrate invariant representation learning with uncertainty-aware modeling and adaptive descriptor strategies for robustness enhancement when new chemical families are encountered. In brief, the results underscore that domain-aware validation and representation-level invariance constitute necessary steps toward dependable cross-family toxicity prediction under real-life TP scenarios.

## Supplementary Information

Below is the link to the electronic supplementary material.

Supplementary Material 1

## Data Availability

The datasets used and/or analysed during the current study available from the corresponding author on reasonable request.

## List of symbols

ML: Machine learning
IID: Independently and identically distributed
QSAR: Quantitative structure–activity relationship
NIH: National Institutes of Health
NCATS: National Center for Advancing Translational Sciences
MGE: Molecular graph encoding
K: Number of clusters
LOCO: Leave-one-cluster-out
R^2^: Coefficient of determination
RMSE: Root mean squared error
MAE: Mean absolute error
r: Pearson correlation coefficient
R^2^_within: Coefficient of determination on seen (training) domains
R^2^_unseen: Coefficient of determination on unseen (test) domain
MW: Molecular weight
NRB: Number of rotatable bonds
nDB: Number of double bonds
IRM: Invariant risk minimization
